# The effects of *Staphylococcus aureus* biofilm conditioned media on 3T3 fibroblasts

**DOI:** 10.1093/femsmc/xtab010

**Published:** 2021-08-16

**Authors:** Leigh Madden, Shyan Huey Low, Anthony R J Phillips, Kimberly A Kline, David L Becker

**Affiliations:** Lee Kong Chian School of Medicine, Nanyang Technological University, Clinical Sciences Building, 11, Mandalay Road, Singapore, 308232; Skin Research Institute Singapore, Level 17, Clinical Sciences Building, 11, Mandalay Road, Singapore, 308232; Lee Kong Chian School of Medicine, Nanyang Technological University, Clinical Sciences Building, 11, Mandalay Road, Singapore, 308232; Department of Surgery, School of Biological Sciences, Auckland University, Symonds street, Auckland Central, New Zealand, 1010; School of Biological Sciences and Singapore Centre for Environmental Life Sciences Engineering (SCELSE), Nanyang Technological University, 60 Nanyang Drive, Singapore 637551; Lee Kong Chian School of Medicine, Nanyang Technological University, Clinical Sciences Building, 11, Mandalay Road, Singapore, 308232; Skin Research Institute Singapore, Level 17, Clinical Sciences Building, 11, Mandalay Road, Singapore, 308232

**Keywords:** *Staphylococcus aureus*, wound healing, fibroblasts, chronic wounds, infection, biofilm

## Abstract

*Staphylococcus aureus* (SA) is the most common bacterial species in chronic wounds. However, there is a lack of understanding of how SA secretions affect the cell biology during the healing process. We studied the effects of biofilm-secretions from SA strain SA29213 on 3T3 fibroblasts. SA29213 is a chronic wound isolate and widely used as a reference strain. We used a series of concentrations of biofilm-conditioned media (BCM) and found 100% BCM is lethal within 10 h. Cells survived in ≤75% BCM but the rate of closure in scratch wound assays was reduced. Treatment with 75% and 50% BCM caused fibroblasts to change shape and develop dendrite like processes. Prolonged treatment with 75% and 50% BCM reduced cell proliferation and increased the 4n deoxyribonucleic acid cell population with cell cycle arrest. There was also an elevation in the senescence marker beta galactosidase and the number of multinucleated cells. Shorter treatments with 75% and 50% SA BCM caused an increase in cell–cell adhesion and a redistribution of β-catenin from the cell membrane to the cytoplasm along with a change in the appearance and decrease in size of ZO-1, vinculin and paxillin structures. Fibroblasts in the edge of chronic wounds exposed to the secretions of SA may suffer similar effects such as induction of senescence, reduced proliferation and migration, which may contribute to the delayed healing of these chronic infected wounds.

## Teaser

SA biofilm conditioned media affects fibroblasts by causing cell cycle arrest, reduction in junctional protein expression, induction of senescence and inhibition of cell migration in scratch wound assays.

## INTRODUCTION

Wound healing involves a series of overlapping processes that include haemostasis, inflammation, cell proliferation, migration, contraction of the wound and remodeling (Martin and Nunan 2015). Normally this proceeds rapidly and without incident. However, in diabetics and elderly individuals with poor vasculature, the process can stall and wounds remain open for months or years (Nunan, Harding and Martin 2014). Chronic wounds, such as diabetic foot ulcers, pressure ulcers and venous leg ulcers, are often locked in a pro-inflammatory state (Menke *et al*. 2007) and fail to heal (Stadelmann, Digenis and Tobin 1998). Chronic wounds show a reduction in extracellular matrix compared to a normal, acute wound (Herrick *et al*. 1992), contain fibroblasts that display signs of senescence with reduced proliferation and migration (Mendez *et al*. 1999; Wall *et al*. 2008), and often have bacterial infections. Bacterial biofilms have a polysaccharide-based extracellular matrix and are often resistant to antibiotics (Edwards and Harding 2004). These biofilms excrete a variety of proteins, peptides, lipids and small molecule metabolites, which can have negative effects on mammalian cells (Bowler, Duerden and Armstrong 2001). Approximately 60–78% of chronic wounds contain bacterial biofilm (Malone *et al*. 2017) (compared to only 6% of acute wounds (James *et al*. 2008)) with *Staphylococcus aureus* (SA) being the most common species (Gardner *et al*. 2004). Biofilms are increasingly considered to be a major contributing factor in perturbing the normal healing of wounds, resulting in chronic wounds (Bowler, Duerden and Armstrong 2001; Edwards and Harding 2004; James *et al*. 2008; Siddiqui and Bernstein 2010). SA and *Pseudomonas aeruginosa* biofilms have been shown to significantly delay wound healing, reducing the rate of reepithelialization and granulation tissue formation (Roche *et al*. 2012; Pastar *et al*. 2013). Currently, the most effective treatment for biofilm-infected wounds is to perform frequent debridement to remove it (Wolcott, Kennedy and Dowd 2009). However, biofilm infected wounds can be hard to identify, as they can appear similar to slough or debris in the wound bed (Schultz *et al*. 2017). There is a clear need to better understand how biofilm secretions alone, rather than the combination of secretions and bacterial coat proteins, can affect the cells around them in relation to the wound healing process.

The effects of bacterial biofilm secretions on *in vitro* wound healing responses have been studied by comparing effects of planktonic-conditioned media (PCM) and biofilm-conditioned media (BCM) on fibroblasts or keratinocytes (Kirker *et al*. 2012; Xu *et al*. 2021). There remains a lack of detailed understanding regarding what elements of the cell biology of wound healing processes are affected. Here we investigated the effects of SA BCM secretions on fibroblasts in *in vitro* assays and find that several different aspects of the wound healing process are affected. SA BCM (75% and 50% BCM, as might be found adjacent to bacterial biofilms) induced cell cycle arrest, senescence, reduced cell migration, increased cell-cell adhesion, with loss of focal adhesion contacts. All of these changes in the fibroblasts are likely to have negative effects on the healing process.

## MATERIAL AND METHODS

### Cell culture

NIH 3T3 (mouse embryonic fibroblasts cell line) were grown in Dulbecco's Modified Eagle Medium (DMEM, Sigma-Aldrich) supplemented with 10% fetal bovine serum (FBS, Gibco) and 1% penicillin/streptomycin (P/S, Gibco) in 5% CO_2_ at 37°C.

### Bacterial growth and BCM

SA ATCC 29213 (SA) was grown in tryptic soy broth (TSB, Sigma-Aldrich). To provide a surface for bacterial biofilm formation, a single layer of wide-opened polyester (PE) fleece was used (Schleheck *et al*. 2009). PE fleece was obtained from an aquaria retail and normalized for each flask by pre-cutting to approximately 8.5 cm diameter and individually adjusted to around 1.5 mg. Culture flasks with PE fleece were autoclaved before use.

Single bacterial colony 10 ml pre-culture was grown at 140 rpm 37°C to OD_600nm_ 0.8–1.0. Cultures were diluted to a final OD_600nm_ 0.01 in 100 ml of TSB (500 ml flasks) and shaken at 140rpm 37°C for 24 h. Fresh TSB was replaced after 17 h to maximize bacterial growth. TSB cultures were replaced with DMEM with 10% FBS and left shaking at 140rpm 37°C for another 24 h. A small section of PE fleece was taken from different points to monitor biofilm growth by microscopy and cfu counts. Cultures were spun down to remove bacteria and DMEM from all flasks were pooled and sterile filtered. The collected media is referred to as BCM and was adjusted to pH 7.4 before use.

### MTT assay

Fibroblasts were grown in flat bottom 96 well plates (Falcon), and incubated with 100 µl BCM containing media. At each time point, BCM containing media was removed from cells and replaced with 100 µl of 10% 3-(4,5-dimethylthiazol-2-yl)- 2,5-diphenyl tetrazolium bromide (MTT, Millipore), diluted in complete DMEM and incubated at 37°C. After 24 h, the media was carefully removed, and MTT formazan was dissolved in 100 µl of isopropanol with 0.04 N HCl. Plates were incubated for another 24 h before absorbance was measured. The absorbance at 570 nm was measured on an ELx800 Absorbance Microplate Reader (BioTek) using Gen5 plate reading software. Results were quantified as a percentage of the control's absorbance.

### Scratch wound assay and propidium iodide uptake

Fibroblasts were plated on a 24 well plate, seeded at 10^5 cells per ml (Thermo Scientific) the day before the experiment and allowed to reach full confluence. A scratch wound was generated with a yellow pipette tip and cells were washed once with fresh complete media containing 1 μg/ml Hoechst before setting up points for imaging. Prior to imaging, the media containing Hoechst (Sigma) was replaced with complete media or media with different dilutions of BCM containing 1 μg/ml of propidium iodide (PI) and 1 μg/ml Hoechst. Time-lapse imaging was performed in an incubation chamber at 37°C in presence of 5% CO_2_ with a Leica DMI 6000. The distances migrated were analyzed with Image J software (NIH) plugin by manually drawing the leading edge of each time point. Conversion of migration distance from pixels to μm was performed. Image J software (NIH) plugin was also used to determine the total number of cells (Hoescht positive) and total number of dead cells (PI positive) in each image. At least two areas each (wound and non-wound) were taken for each well and three technical replicates for each experiment was performed.

### Cell count

Fibroblasts were grown in 24 well plates in BCM or DMEM supplemented with 10% FBS and 1% PS. At the indicated time points, cells were trypsinised and re-suspended in media and trypan blue. The number of live cells in an aliquot was counted manually using a counting chamber.

### Senescence assay (β-galactosidase activity)

Fibroblasts were grown on an eight well chamber cover glass (Labtek) with BCM and media change to fresh BCM every 2–3 days. To stain cells for β-galactosidase activity, senescent cells histochemical staining kit (Sigma) was used. Cells were fixed with 1 x fixation buffer, washed with PBS and staining mixture was added and incubated at 37°C for 24 h without CO_2._ Cells were then washed with Phostphate Buffered Saline (PBS) and stained with 4',6-diamidino-2-phenylindole (DAPI) before being imaged. At least five regions were imaged per well.

### Flow cytometry

Fibroblasts were collected by trypsinisation, washed with ice-cold PBS and spun down to attain a pellet. Cells were fixed with ice-cold absolute ethanol, washed with PBS and stained with phospho-histone H3 (Ser10) Rabbit mAb 488 conjugate (cell signaling 3465, 1:50) for 1 h at room temperature. Cells were washed with PBS and incubated with PI and ribonuclease. Samples were analyzed on a flow cytometer.

### Adhesion/hanging drop assay

Approximately 20 000 single cells were suspended in 35 µl drops of 10% FBS in DMEM in wells of 24-well plate. PBS was placed as drops on the lids of the plate to maintain humidity. The plate was inverted during the experiment. After 4 h, the plate was reinverted and the drops in the well were pipetted five times with 200 μl yellow tip, and drops were spread out with a coverslip on top. For each biological replicate, at least six random fields from three samples were taken. The area of each cell cluster was determined using a custom-written plug-in for ImageJ software (NIH).

### Immunofluorescence and confocal imaging

Fibroblasts were grown on 24 well plates with glass coverslips in each well. After 4 h of BCM treatment, cells were fixed with 4% paraformaldehyde (PFA) for 10 min, permeabilized 10 min with 0.1% Triton X-100 (Sigma) and 30 min blocking with 0.1 M L-lysine monohydrochloride in PBS. Samples were incubated with Cx43 (Sigma C6219, 1:4000), ZO-1 (Invitrogen 339 100, 1:100), β-catenin (Abcam ab6302, 1:2000), α-tubulin (Sigma T6074, 1:1000), GM130 (BD transduction Laboratories 610 822, 1:200), phospho-Histone H3 (Ser10) (Milipore 09–797, 1:1000), vinculin (Sigma) and paxillin (Abcam) at room temperature for 1 h. Samples were washed with PBS, incubated with secondary antibodies for 1 h at room temperature, stained with DAPI and/or rhodamine-phalloidin, washed in PBS and mounted in Citifluor® (Glycerol/PBS solution, Citifluor Ltd). Immunofluorescence was imaged on a Leica SP8 upright confocal microscope. Optimal gain and offset were set and kept constant during image acquisition. Minimum of three regions were imaged of each biological replicate.

### Quantification of immunofluorescence

Confocal images were imported into ImageJ (NIH) software to perform quantification. The 8-bit images were thresholded identically. The total area of connexin expression was calculated and then normalized to the number of nuclei in each image. For ZO-1, vinculin and paxillin, average particle size was calculated and normalized to controls. The results from experimental replicates were averaged.

### Statistical analysis

This was performed using an analysis of variance (ANOVA) followed by a Tukey test. A value of <0.05 was considered significant (* *P* <0.05, ** *P* <0.01, *** *P* <0.001, ^****^P <0.0001).

## RESULTS

### SA BCM at high concentrations can reduce metabolic activity and cell viability

**Figure 1.**
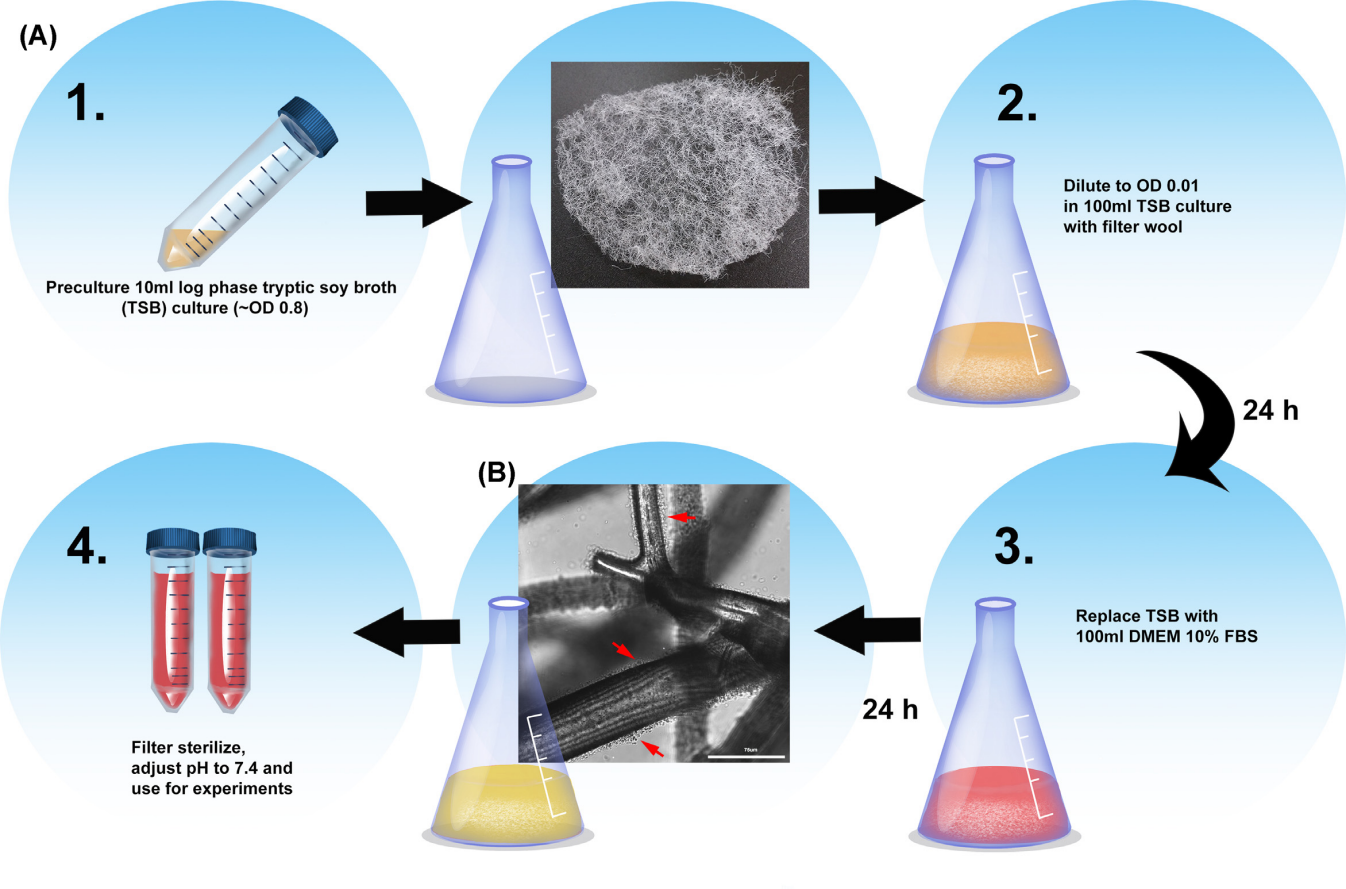
BCM production. **(A)** Workflow of BCM production. **(B)** A representative image of an area of filter wool with SA growth as a biofilm layer before BCM collection. Scale bar represents 75 μm.

**Figure 2.**
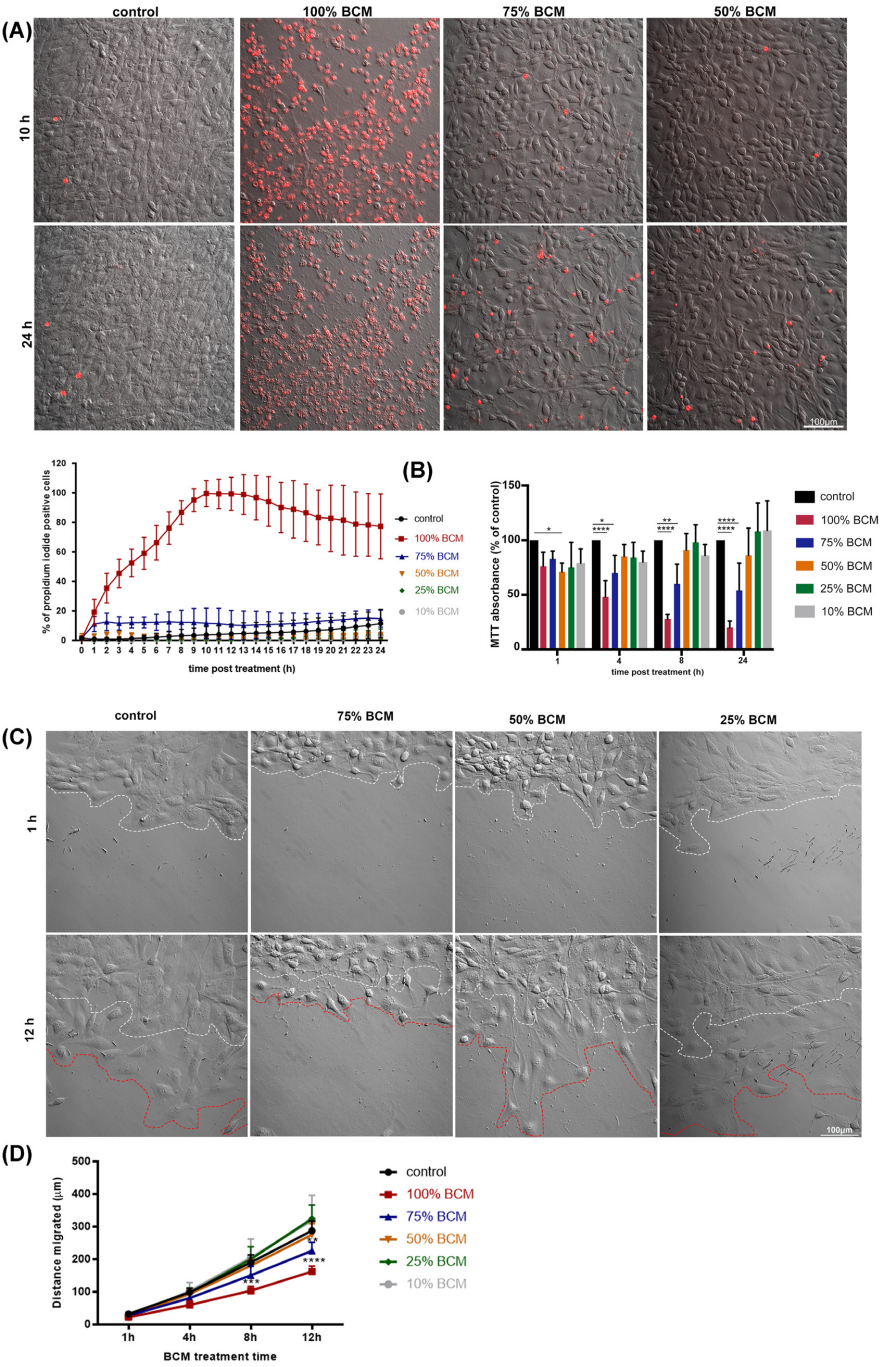
BCM at high concentrations reduces metabolic activity, cell viability and reduces cell migration. **(A)** Representative images of 3T3 fibroblast treated with different dilutions of BCM at 10 h and 24 h post treatment. PI (red) live dead stain was used to determine percentage of dead cells. Scale bar represents 100 μm. Graph shows percentage PI positive cells. Data are shown as mean ± standard deviation (SD) (n = 3/4). **(B)** MTT assay shows percentage absorbance of metabolically active cells upon treatment. Cells were treated with BCM for respective time points before MTT was added for 24 h. **(C)** Images show 3T3 fibroblast scratch wound edge at 1 h (white dotted line) and 12 h (red dotted line) post BCM treatment. Scale bar represents 100 μm. **(D)** Graph shows distance migrated by 3T3 fibroblasts with different dilutions of BCM treatments. Data are shown as mean ± SD (n = 3–5); two-way ANOVA followed by Tukey post hoc analysis; * *P* <0.05, ** *P* <0.01, *** *P* <0.001, ^****^P <0.0001.

To study the effects of SA biofilm secretions on fibroblasts and their wound healing responses, large batches of SA BCM were produced (Fig. 1). To test the effects of different concentrations of BCM on fibroblast viability, we performed time lapse imaging with PI in the media. PI is excluded from live cells but as cells and their membranes become compromised and PI enters the cell labeling the nuclei red. This allows the percentage of viable cells to be determined. We found that 100% BCM killed most of the cells within 10 h of treatment (Fig. 2A). Lower concentrations of BCM (75% and below) caused markedly less cell death, and were indistinguishable from untreated controls. To understand how the metabolic activity of fibroblasts is affected by SA BCM, we performed an MTT assay. Treating cells with 75% BCM caused a significant reduction in fibroblast metabolic activity between 1 h and 24 h (*P* <0.05–*P* <0.0001). In contrast, 50% SA BCM produced only a slight decrease in metabolic activity (Fig. 2B); no effect on metabolic activity was detected with 25% and 10% SA BCM.

The effects of SA BCM on a scratch wound assay were examined (Fig. 2C) over 12 h and there was a significant (*P* <0.05–*P* <0.0001) decrease in distance migrated by cells following 75% BCM treatment (Fig. 2D). The morphology of the cells changed and had lost their ability to form lamellipodia. However, there was no difference in migration rate following 50% BCM treatment.

**Figure 3.**
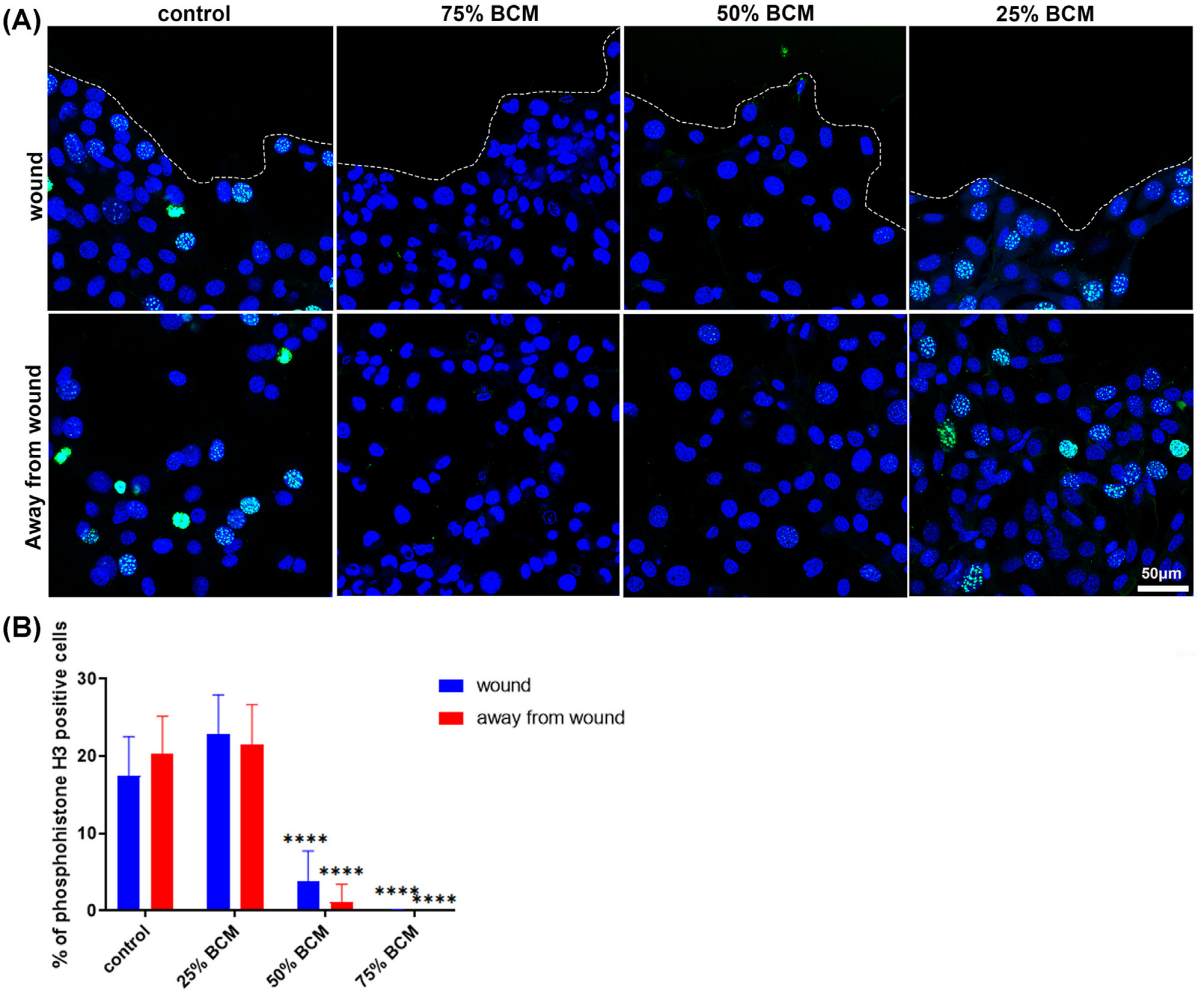
High concentrations of BCM reduce cell proliferation. **(A)** Images show 3T3 fibroblasts at areas of scratched wound edge (white dotted line) or away from wound 4 h post BCM treatment. Cells were stained with phosphohistone H3 (green) and DAPI (blue) to determine number of proliferating cells. Scale bar represents 50 μm. **(B)** Graph shows percentage of phosphohistone H3 positive cells. Data are shown as mean ± SD (n = 3–5) two-way ANOVA followed by Tukey post hoc analysis; ^****^P <0.0001.

To better understand the effects of SA BCM on cell proliferation, cells treated with different concentrations of BCM were stained for phosphohistone-H3. A 4-h treatment with 75% and 50% BCM significantly (*P* <0.0001) reduced the number of phosphohistone-H3 positive, actively proliferating cells both at the wound edge and away from the wound. In contrast, 25% BCM had no effect on cell proliferation (Fig. 3).

### Prolonged treatment with SA BCM induces cell cycle arrest and senescence

**Figure 4.**
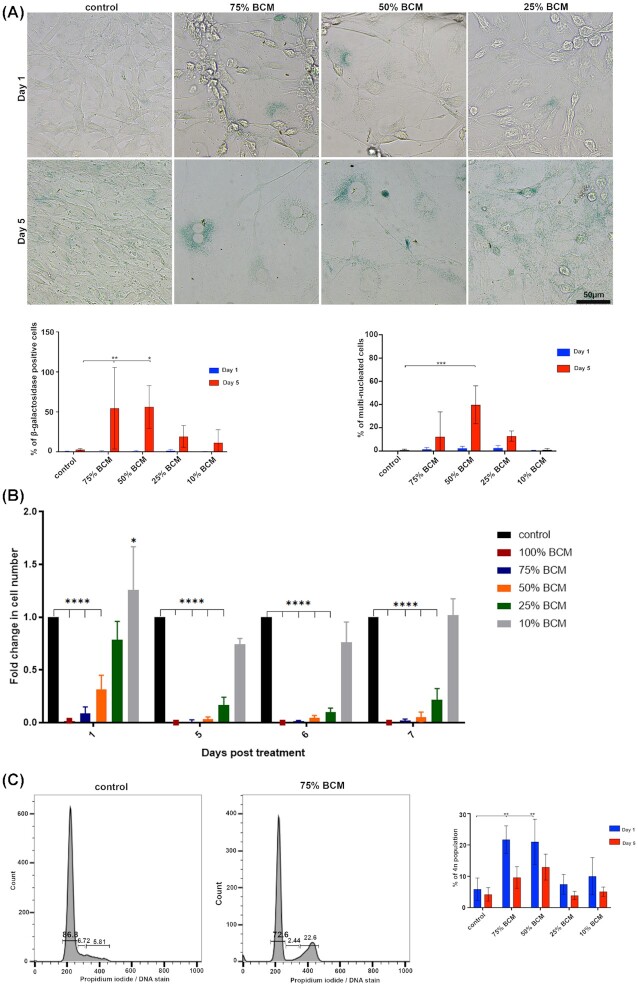
Prolonged incubation (5 days) with BCM leads to cell senescence. **(A)** Images show 3T3 cells at day one and five post treatment. Cells were stained with a senescence kit to detect senescent cells with β-galactosidase activity (blue). Graph shows percentage of β-galactosidase positive cells day one and day five upon BCM treatment. Cell media was replaced with fresh BCM every 2–3 days. Data are shown as mean ± SD (n = 3/4); two-way ANOVA followed by Tukey post hoc analysis; ** *P* <0.01, * *P* <0.05. Graph shows percentage of multinucleated cells. Cells were replaced with fresh BCM every 2–3 days. Data are shown as mean ± SD (n = 3/4); two-way ANOVA followed by Tukey post hoc analysis; *** *P* <0.001. **(B)** Graph shows viable cell count of 3T3 fibroblasts at day one, five, six or seven post BCM treatment. Trypan blue was added to the cells before counting, with only cells negative for trypan blue counted as viable cells. Data are shown as normalized to the control, with mean ± SD (n = 3). * *P* <0.05, ^****^P <0.0001. **(C)** 3T3 fibroblasts upon BCM treatment were stained with PI, which binds to DNA and allows for quantitative DNA content or cell cycle analysis with flow cytometry. The graph shows three population of cells with 2n (G1 phase, resting non dividing cells), 2n-4n (S phase, DNA replicating cells) and 4n (G2/M phase, cells that finished replication and awaiting division). Graph shows % of 4n population day one or day five post BCM treatment. Data are shown as mean ± SD (n = 3); two-way ANOVA followed by Tukey post hoc analysis; ** *P* <0.01. Scale bar 50 μm.

As biofilms can persist in wounds for days or weeks, we examined the effects of prolonged BCM treatment on fibroblasts for up to 7 days then stained them for beta-galactosidase, a marker of senescence. Cells treated with 75% BCM and 50% BCM expressed little, if any, beta-galactosidase at day one but this increased significantly (*P* <0.05–*P* <0.001) by day five to seven (Fig. 4A). Cells at these later timepoints showed a flattened morphology, with an enlarged cell body containing lipofuscin vesicles around the nucleus, and an increased frequency of multinucleated cells (Fig. 4A). Treatment with 75% and 50% SA BCM for one day led to a significant (*P* <0.01) increase in the number of cells with 4n deoxyribonucleic acid (DNA) as assessed by PI staining and flow cytometry, indicating cell cycle arrest at G2M (Fig. 4C). Cells treated with 25% BCM and controls showed no increase in 4n DNA.

### SA BCM affects cytoskeletal proteins and increases cell-cell adhesion

**Figure 5.**
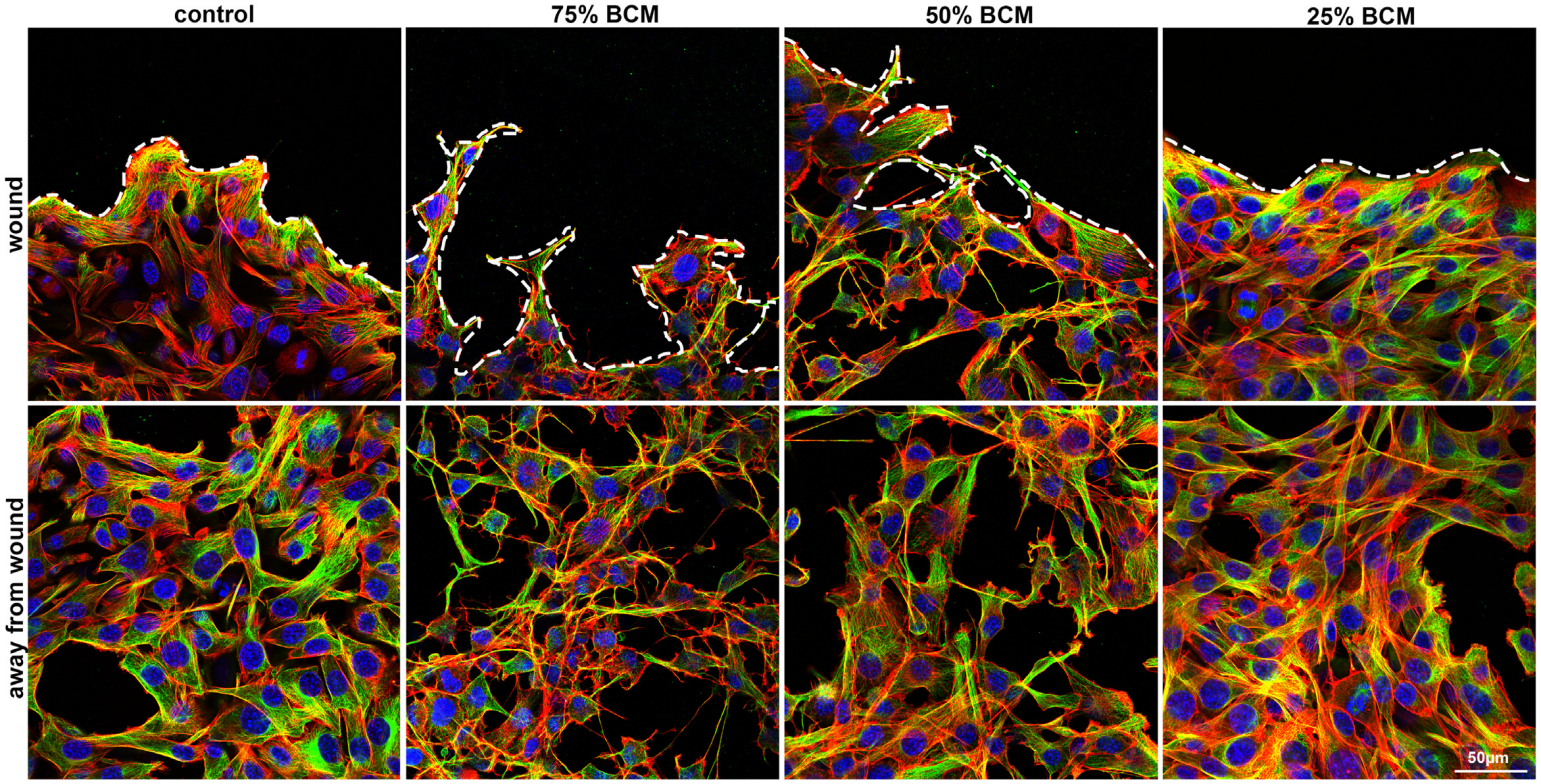
BCM changes cell morphology of 3T3 fibroblasts both at and away from the wound. Images show 3T3 fibroblasts at and away from the wound. Cells were stained with DAPI for nucleus (blue), phalloidin for actin cytoskeleton (red) and α-tubulin (green). Scale bar represents 50 μm.

To examine the effects of SA BCM on the cytoskeletal proteins F-actin and alpha-tubulin, we immunostained cells in scratch wound assays 4 h post scratch and SA BCM treatment. In the control, fibroblasts at the wound edge had elevated levels of tubulin compared with those distal to the wound (scratch; Fig. 5). Actin stress fibers were aligned with the wound edge in the leading-edge cells but were evenly distributed throughout the cell body in cells further away. The 25% BCM-treated cells showed a similar morphology and distribution of actin and tubulin to control cells. Similarly, cytoskeletal dynamics were normal.

When treated with 75% and 50% BCM, fibroblast morphology changed in cells both at the wound edge and further away. These fibroblasts had contracted cell bodies, into a neuronal-like appearance, with elongated cellular extensions that stained strongly for F-actin and tubulin. At the wound edge, there was no clear elevation of tubulin in 75% BCM or 50% BCM compared to control. The normal changes in cytoskeletal dynamics as part of the wound healing response were severely perturbed following 75% and 50% BCM treatment, compromising the healing process.

**Figure 6.**
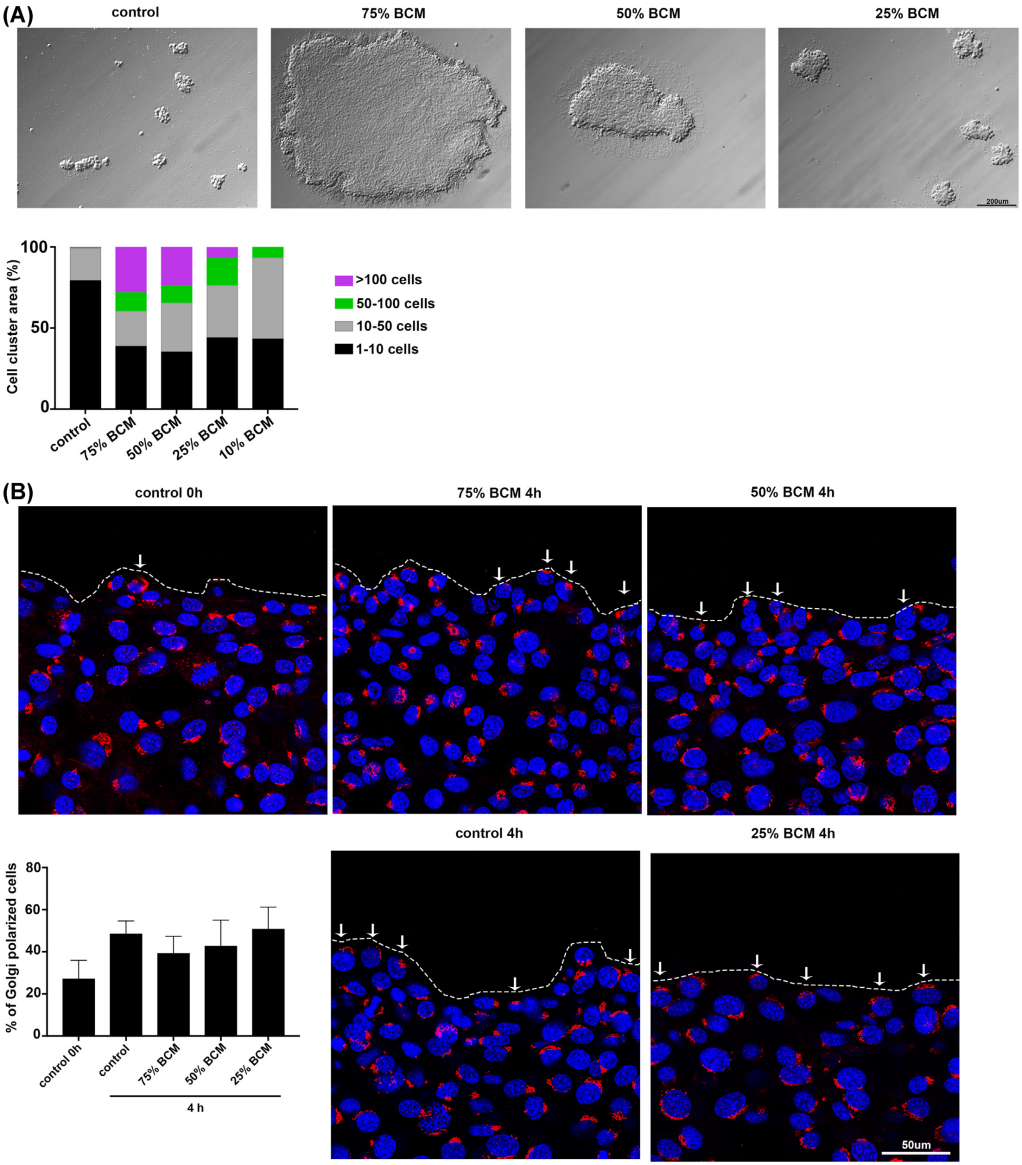
BCM increases cell-cell adhesion in dose dependent manner with no effect on cell polarization. **(A)** Images show 3T3 fibroblasts in hanging drop assay after treatment with BCM for 4 h and trituration for 10 times. Scale bar 100 μm. (A) Quantification of cell cluster area of different BCM treatments. Area of cells was determined manually by drawing around each cluster with ImageJ and number of cells was determined by correlating to the area of one cell. **(B)** Images show cells at 0 h or 4 h post BCM treatment stained with GM130 (red) for Golgi and DAPI (blue) for nucleus. White line represents wound edge and white arrows show cells that have polarized Golgi towards the wound edge. Scale bar represents 50 μm. Graph shows quantification of percentage of Golgi polarized cells which was performed manually.

To study the effects of BCM on cell-cell adhesion, we performed a hanging drop assay. Cells were incubated with BCM for 4 h in a hanging drop of media, then triturated 10 times with a yellow pipette tip. A BCM concentration-dependent increase in cell-cell adhesion and larger clumps was observed upon treatment (Fig. 6A). The 75% and 50% BCM had bigger clumps of more than 100 cells. This suggests that BCM increases cell-cell adhesion in a concentration-dependent manner.

To monitor changes in repolarization of leading-edge cells 4 h post BCM treatment, we stained them with a GM130 antibody for the microtubule organizing center (MTOC). There was more MTOC repolarization at the leading edge of the control cells at 4 h post treatment compared to 0 h. However, fibroblasts treated with all concentrations of BCM still managed to repolarize their MTOC in the wound edge cells 4 h post scratch wounding.

### BCM alters appearance and size of junctional and adhesion proteins in fibroblasts

**Figure 7.**
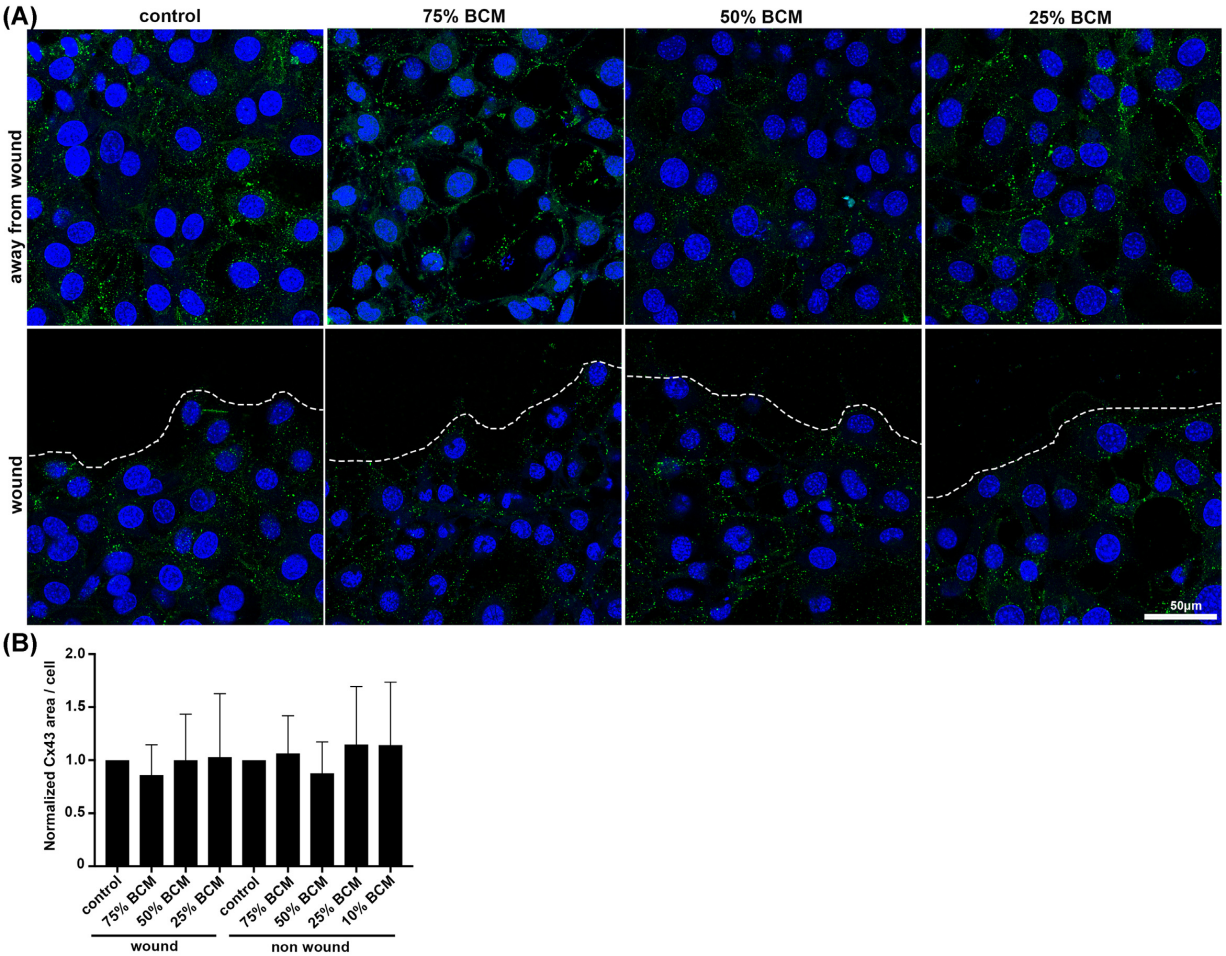
Cx43 levels were not affected by BCM treatment. **(A)** Images show Cx43 (green) and DAPI (blue) staining at and away from scratch wound with different BCM treatments. Scale bar 50 μm. **(B)** Quantification of Cx43 area/cell shows no significant differences.

**Figure 8.**
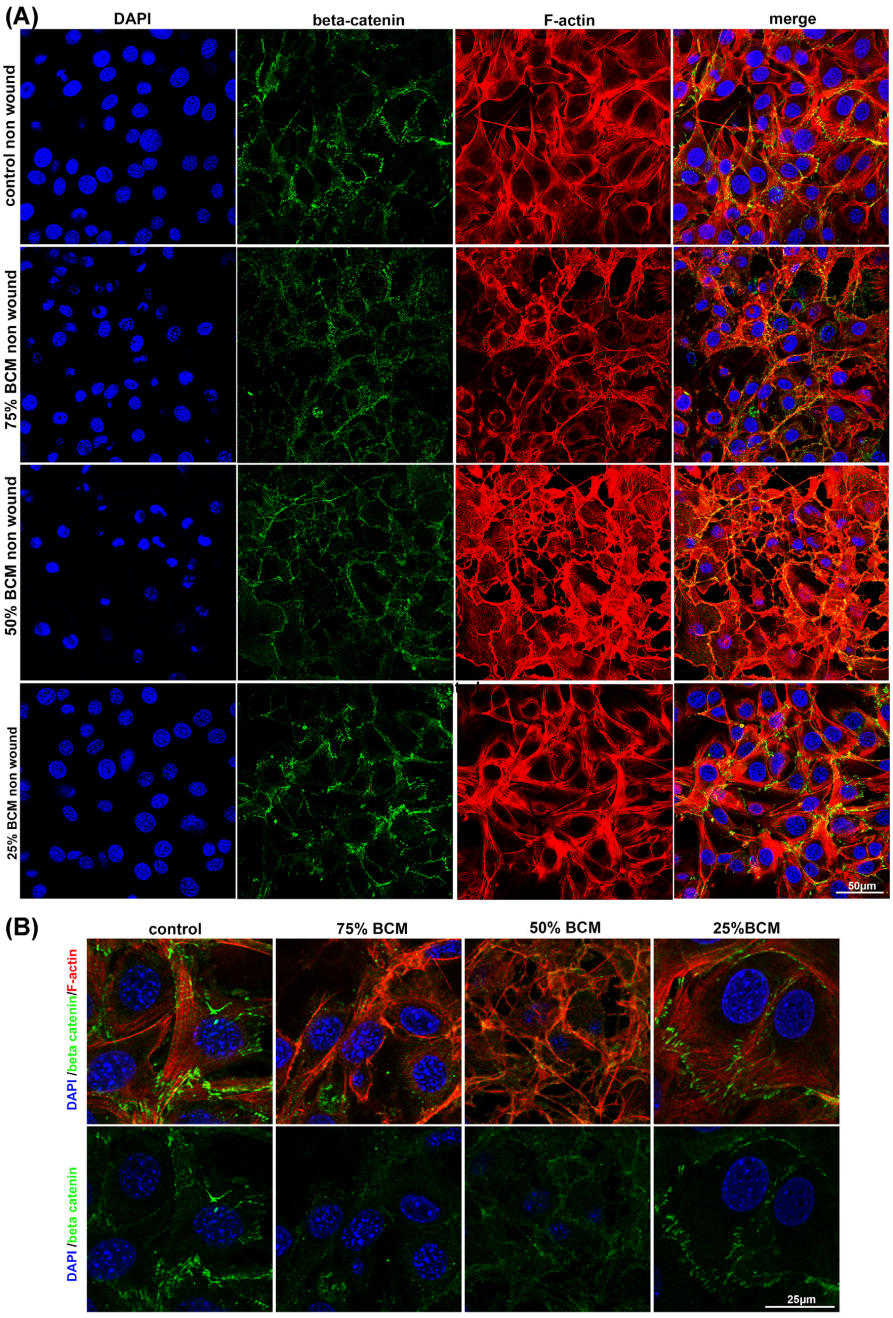
BCM cause beta catenin to relocate from the cell membrane to the cytoplasm. **(A)** Images show cells stained with beta catenin (green), DAPI for nucleus (blue) and F-actin (red). Scale bar represents 50 μm. **(B)** Higher-power images of stained cells. Scale bar represents 25 μm.

**Figure 9.**
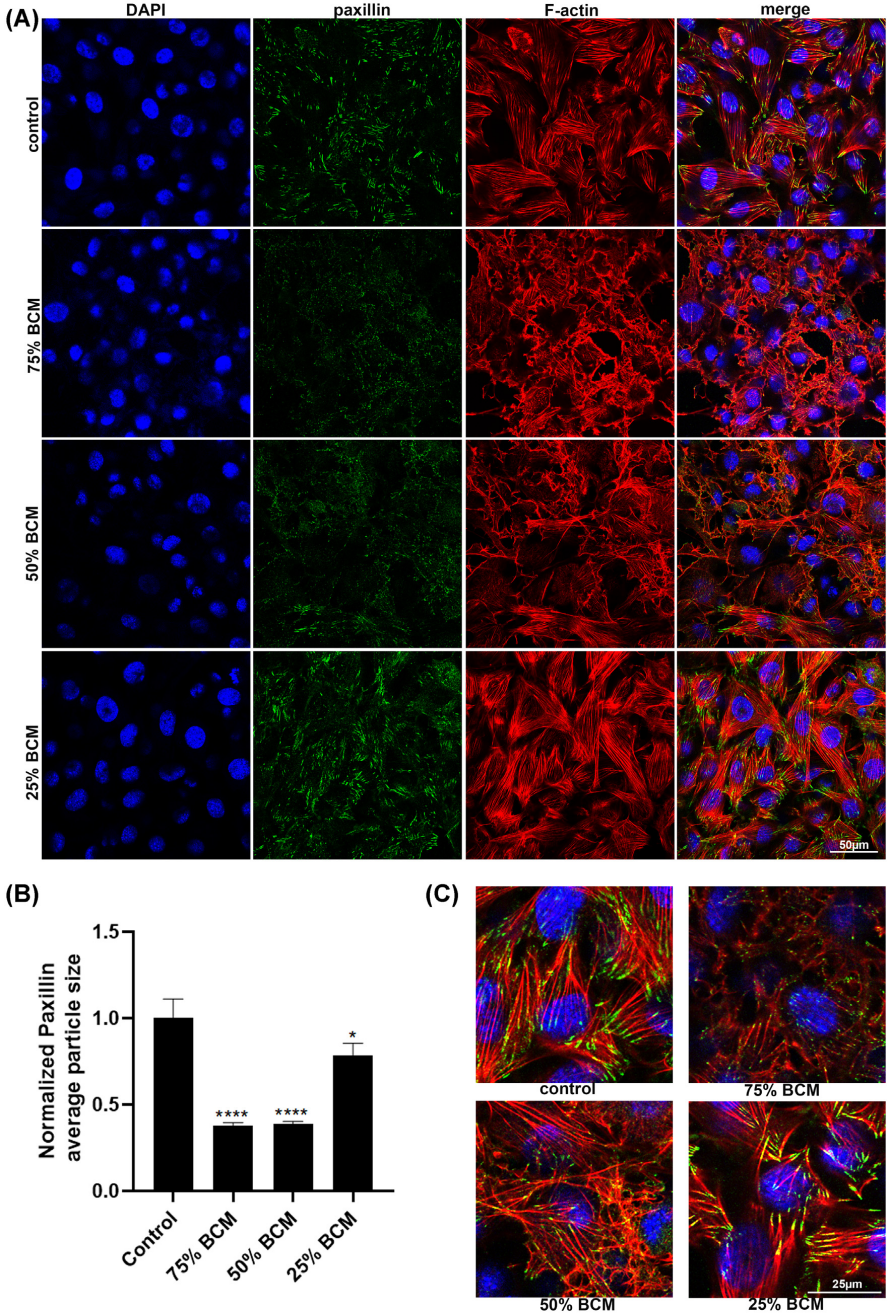
BCM effects the appearance and reduces the size ofpaxillin structures between cells and the plastic dish. Images show cells stained with paxillin (green), DAPI for nucleus (blue) and F-actin (red). Scale bar represents 50 μm. **(B)** Quantification of average paxillin particle size shows significant differences of BCM treated cells vs control. (* *P* <0.05, ^****^ *P* <0.0001) **(C)** Higher-power images of paxillin stained cells. Scale bar represents 25 μm.

**Figure 10.**
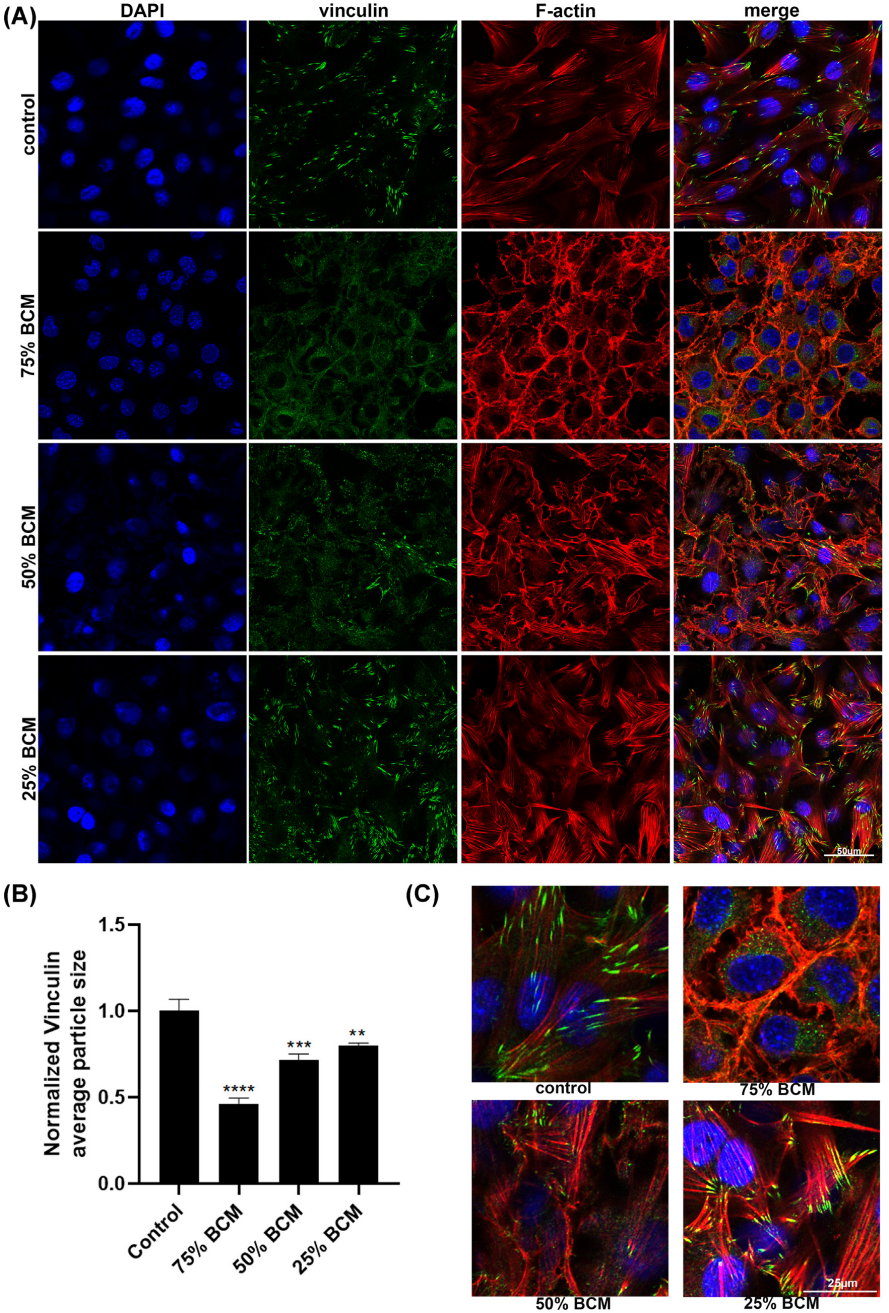
BCM effects the appearance and reduces the size of vinculin structures between cells and plastic dish. Images show cells stained with vinculin (green), DAPI for nucleus (blue) and F-actin (red). Scale bar represents 50 μm. **(B)** Quantification of average vinculin particle size shows significant differences of BCM treated cells vs control. (* *P* <0.05, ** *P* <0.01, *** *P* <0.001, ^****^P <0.0001) **(C)** Higher-power images of vinculin stained cells. Scale bar represents 25 μm.

**Figure 11.**
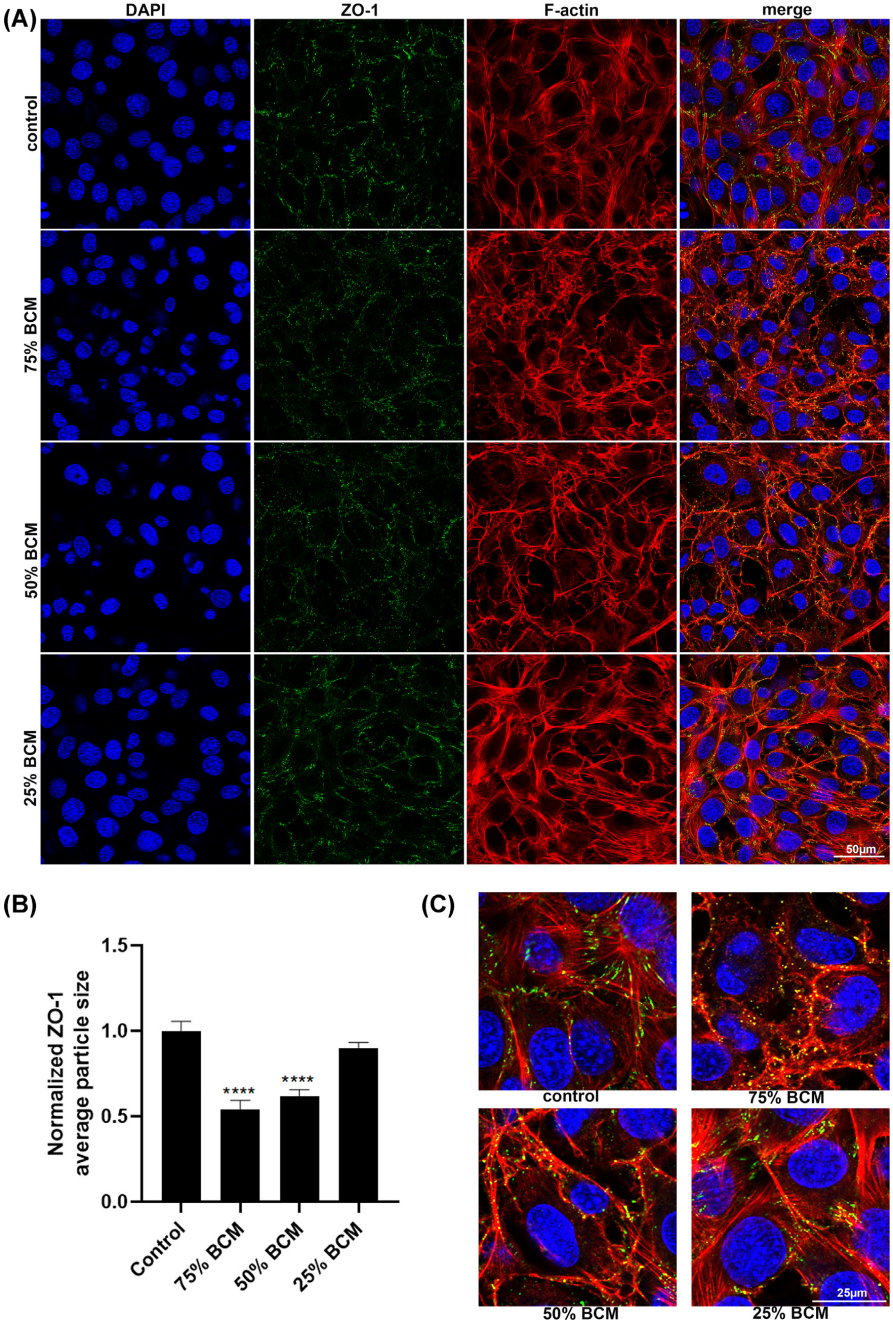
BCM effects the appearance and reduces the size ofZO-1structures, from elongated to punctated on the membrane. **(A)** Images show cells stained with ZO-1 (green), DAPI for nucleus (blue) and F-actin (red). Scale bar represents 50 μm. **(B)** Quantification of average Z0-1 particle size shows significant differences of BCM treated cells vs control. (* *P* <0.05, ** *P* <0.01, *** *P* <0.001, ^****^P <0.0001) **(C)** Higher-power images of ZO-1 stained cells. Scale bar represents 25 μm

We stained for different junctional or adhesion protein antibodies in cells treated for 4 h with BCM. As Cx43 is upregulated in human chronic wounds, and is known to inhibit their cell migration, we examined Cx43 levels in SA BCM treated fibroblast cells (Mendoza-Naranjo *et al*. 2012). There were no significant changes in Cx43 protein levels with any concentrations of BCM treated fibroblasts (Fig. 7). Staining for beta-catenin showed that it was relocated from the cell membrane to the cytoplasm in cells treated with 75% and 50% BCM (Fig. 8). We observed a reduction in average size of the focal adhesion proteins vinculin and paxillin (Figs 9 and 10) at the contact points between cells and the plastic dish with BCM treatment, suggesting BCM is affecting cell-substrate attachment. Staining of treated cells for ZO-1, a tight junction protein, showed a significant decrease in ZO-1 protein structure size following 75% BCM treatment. ZO-1 appeared to be more punctate rather than the linear bands seen in control cells (Fig. 11).

## DISCUSSION

The observed reduction in cell viability after treatment with high concentrations of BCM had been previously reported in a variety of cell types, including fibroblasts (Kirker *et al*. 2012), keratinocytes (Kirker *et al*. 2009; Tankersley *et al*. 2014), osteoblasts (Sanchez *et al*. 2013; Cassat *et al*. 2013) and macrophages (Scherr *et al*. 2015). Investigators used different assays and/or SA strains suggesting that the toxic effects are not SA strain-dependent or cell specific. Most assays performed in such studies were colorimetric and dependent on conversion of a substrate. As such, they did not directly study whether the cells were viable or just metabolically less active.

We found that the metabolic activity of cells treated with 75% BCM significantly reduced within 4 h of treatment; lower doses did not have an effect on metabolic activity, even after longer treatment of 24 h (Fig. 2B). Concentrations of ≤75% BCM were gradually more toxic over a prolonged period (5–7 days) rather than rapidly killing cells this may be the mechanism of death of chronic wound edge cells. It may also be a contributing mechanism, which results in progressive increase in size of some chronic wounds.

Directional migration and cell proliferation are key events in the wound healing process. In a scratch wound assay, only high concentration (75%) BCM reduced the rate of fibroblast migration at 24 h post treatment. This is similar to the effect of PCM and BCM on keratinocyte migration in scratch wound assays (Kirker *et al*. 2009). As MTOC repolarization occurred at all BCM concentrations at same time, this is not likely to be a key factor in delayed migration. In contrast to our findings, greater BCM effects on cell migration in scratch wound assays have been reported for fibroblasts (Kirker *et al*. 2012; Kshetri, Brennan and Vaughan 2016) and keratinocytes (Kirker *et al*. 2009; Jeffery Marano *et al*. 2015; Xu *et al*. 2021). These differences may be due to the BCM concentrations used for assessment. We titrated our BCM due to the cell toxicity at 100%, whereas as the BCM was not titrated in these studies. Marano et al. reported that BCM was not toxic to cells but generated a significant reduction in keratinocyte migration (Jeffery Marano *et al*. 2015). This difference may be due to keratinocytes being more sensitive to BCM than fibroblasts or may be the result of SA strain used.

We observed a significant reduction in the number of cells that were undergoing mitosis. This is consistent with previous reports of BCM reducing proliferation of keratinocytes (Jeffery Marano *et al*. 2015) and osteoblasts (Sanchez *et al*. 2013). Prolonged treatment with higher concentrations of BCM (75% and 50%, up to 7 days) did not show an increase in cell numbers, suggesting that cells that remained present and viable but did not proliferate. Many of the cells had indicators of senescence e.g. enlarged cell bodies, increased beta-galactosidase activity and were multi-nucleated, which were not observed with lower BCM concentrations. Induction of cellular senescence can negatively impact migration. As early as 1 day post treatment with 75% and 50% SA BCM, there was an increase in 4n DNA population. Consistent with our findings, live SA has been reported to delay G2/M transition, enlarge cell bodies and reduce cell proliferation in a variety of epithelial cell lines (Alekseeva *et al*. 2013). Here we show that bacterial biofilm secretions alone, in the absence of live bacteria and their coat components, are sufficient to induce senescence in fibroblasts. High levels of senescent fibroblasts have been found in chronic wounds such as venous leg ulcers (Agren *et al*. 1999). It has been suggested that wounds will not heal in the presence of more than 15% senescent fibroblasts in chronic wound edges (Harding, Moore and Phillips 2005). Our findings suggest that biofilms, which are prevalent in chronic wounds, may inhibit the healing by inducing senescence in fibroblasts.

Bacteria can influence the cell cytoskeleton to invade or survive in the host (Mostowy and Shenoy 2015). BCM, but not planktonic conditioned media, cause changes in cell morphology of keratinocytes to form dendrite-like extensions in wound healing assays (Kirker *et al*. 2009). The reduced rates of scratch wound closure suggest that bacterial biofilm secretions alone are sufficient to cause deleterious effects on cell morphology and migration. Here we report that BCM modified actin and tubulin cytoskeleton of fibroblasts and observed similar dendrite-like extensions. The loss of actin stress fiber structures in BCM-treated cells treated and shrunken cell bodies. The BCM of SA 29213 contains fructose bisphosphate aldolase (ALDOA) (Kusakabe, Motoki and Hori 1997; Jeffery Marano *et al*. 2015) and epidermal cell differentiation inhibitor (EDIN) (Sugai *et al*. 1992). ALDOA can bind directly to the actin cytoskeleton, modulating its polymerization and lamellipodial formation (Kusakabe, Motoki and Hori 1997; Jeffery Marano *et al*. 2015). EDIN can inactivate Rac and Rho GTPase by ADP-ribosylate activity (Sugai *et al*. 1992) influencing the cytoskeletal activities. These molecules within the BCM could negatively influence cell shape and migration. Interestingly, EDIN has also been reported to cause hyperplasia when injected into mouse skin, which is a common feature of human chronic wounds (Sugai *et al*. 1992; Sutcliffe *et al*. 2015).

The observed BCM concentration-dependent increase in cell–cell adhesion may negatively affect cell migration. Interestingly, we did not observe an upregulation in the gap junction protein Cx43, as seen in chronic wounds (Mendoza-Naranjo *et al*. 2012; Sutcliffe *et al*. 2015). This is in contrast to the observation that SA UAMS-1 exudate does elevate Cx43 in HaCat cells and retards their migration (Xu *et al*. 2021). This may reflect a difference in sensitivity between keratinocytes and fibroblasts or higher virulence of SA UAMS-1. We detected changes in the adherent junction protein beta catenin, focal adhesion proteins vinculin and paxillin as well as tight junctional protein ZO-1 after BCM treatment. Effects of bacteria on junctional proteins have been previously reported. *Pseudomonas aeruginosa* quorum sensing molecule 3O-C(12)-HSL led to disruption of adherens junction protein (E cadherin and beta-catenin) and tight junctional proteins (ZO-1, ZO-3, JAM and occludin), which is linked to changes in phosphorylation levels of these proteins and increased paracellular permeability (Vikstrom *et al*. 2009). This molecule, 3O-C(12)-HSL, can also target IQGAP1 that activates Rho GTPases and leads to changes in the cytoskeleton (Vikstrom *et al*. 2009). In addition, hyperadhesiveness of human vascular endothelial cells (HUVECs) to monocytes and granulocytes, mediated by integrins, was shown in SA-infected HUVECs (Beekhuizen *et al*. 1997). Downregulation of integrin beta 3 and 6 in keratinocytes by *Porphyromonas gingivalis* can cause impairment of cell–cell interaction and migration (Bhattacharya *et al*. 2014). Normal fibroblast migration may be detrimentally impacted by an inability of fibroblasts to attach to the substrate, demonstrated by loss of size of focal adhesion structures and adherent junction protein (beta catenin) and tight junction protein (ZO-1) in our studies. Further transcriptomic studies may provide a clearer view of proteins that are affected by BCM secretions. These in vitro studies provide interesting insights but are limited and as such the impact of BCM should be investigated in an animal wound model.

Our findings suggest several mechanisms by which SA biofilms may inhibit wound healing, including cell cycle arrest, senescence, increase cell-cell adhesion and inhibition of cell migration. Some of these effects, such as cell cycle arrest and induction of senescence, may be irreversible and require debridement for normal healing to take place. As such, the prevention of biofilm infections in chronic wounds should be a key aim in promoting the healing of these wounds.

## FUNDING

This research is supported by the Agency for Science, Technology and Research (A*STAR) under its Industry Alignment Fund—Pre-Positioning Programme (IAF-PP) grant number H1701a0004 Skin Research Institute of Singapore, Phase 2: SRIS@Novena.

## DATA AVAILABILITY

The datasets generated during and/or analyzed during the current study are available from the corresponding author on reasonable request.

## Supplementary Material

xtab010_Supplemental_File
