# Supplementary material for: The effects of Staphylococcus aureus biofilm conditioned media on 3T3 fibroblasts
Source: FEMS Microbes. 2021 Aug 16;2:xtab010. doi: 10.1093/femsmc/xtab010 (PMC10117754; doi:10.1093/femsmc/xtab010)
Supplement: xtab010_Supplemental_File [file xtab010_supplemental_file.docx]

Peer reviews and response FEMSMC-2021-003

Reviewers' Comments to Authors:

Reviewer 1: Anonymous

Comments to the Author
Major comments:
1) The reference list is a bit short and much of the previous literature describing the impact of S. aureus secretions on host healing is not discussed. For example but not limited to the following: https://www.ncbi.nlm.nih.gov/pmc/articles/PMC3721972/ and https://www.ncbi.nlm.nih.gov/pmc/articles/PMC3579943/.

2) Based on the figure lettering, figure 5 appears distorted. This probably was just an issue with the uploading that will be corrected in the publication quality figure. More of an issue with this figure is the fact that it is not easy to appreciate the features that the authors are trying to emphasize in the figure. Maybe if the authors were able to include some form of quantification or at least a few replicates for comparison it would be easier to visualize what the authors are concluding from these data.

3) In figure 7, the “away from wound” staining per cell simply visually looks much higher per cell in the 75% BCM than any other treatment. Are the author’s certain the graph and the images are appropriately corresponding in this figure? If there is no mistake, can the authors find a more representative image to display?

4) In figure 8, I am having trouble observing the phenotype the authors claim exists. Can the authors find a way to quantify this phenotype? Maybe if they did a membrane stain too it would be easier to see in a “merge” that there was more membrane localization in the untreated condition?

5) While I can more easily visually appreciate the claimed phenotype for Figure 9 and even more so for Figure 10 relative to the figure 8 phenotype, a method of quantification for both of these figures would also be appreciated.

6) There is a claimed phenotypic difference in Figure 11 but the graph does not seem to depict any statistically significant differences.


Minor comments:
1) Change scale bar to white in figure 2A for more accessible viewing.
2) Change scale bar lettering to a white font in figure 2C for easier reading.
3) Scale bar missing for figure 3A
4) Scale very difficult to read on figure 4A
5) On page 3 lines 42-45, the authors’ statement about it studying biofilm secretions rather than bacterial coat proteins might need better clarification.
6) There are some grammatical errors such as commas that are not needed and missing commas in many places. I would recommend the authors perform an additional proofreading to this paper and correct these grammatical errors.


Reviewer 2: Dr Magnus Ågren

Comments to the Author
Interesting in vitro results of the effects of S. aureus biofilm products. The limitation of the study should, however, be discussed such as the use of only one target eukaryotic cell (3T3 mouse fibroblasts) and one bacterial strain. Do human cells behave the same? The authors speculate on the possible selective negative effects on wound edge fibroblasts; but why do the authors think that these cells are more sensitive to the deleterious effect of S. aureus biofilm secretions than cells (e.g., fibroblasts) in the wound bed. Finally, the authors should discuss possible factors that are produced by the biofilm causing cell damages.

Specific comments
1. Abstract page 2, line 15: Please specify etiology of the chronic wound S. aureus was derived from.
2. Page 4, line 52, 60: change ul to µl.
3. Page 5, line 12: Please indicate seeding density in the scratch experiment.
4. Page 7, line 18: Please indicate the definition of statistical significance.
5. Page 7, line 46, 56: Present the magnitude of the effects rather than the p values.
6. Page 11, line 33: Please cite the original finding of senescent fibroblasts in venous leg ulcers (PMID: 10201530).
7. The Discussion is very technical, repetitive of results and too long; please shorten and discuss more general, possible factors that are produced by the biofilm causing cell damages and the limitations of the study.

Author Response:

Response to Reviewers comments:
Manuscript ID: FEMSMC-2021-003
Title: The effects of Staphylococcus aureus biofilm conditioned media on 3T3 fibroblasts.
Authors: Madden, Leigh; Low, Shyan; Phillips, Anthony; Kline, Kimberly; Becker, David
Submitted to: FEMS Microbes

We thank the reviewers for their constructive and helpful comments which we have tried to address.
Reviewer: 1
1)The reference list is a bit short and much of the previous literature describing the impact of S. aureus secretions on host healing is not discussed. For example but not limited to the following: https://www.ncbi.nlm.nih.gov/pmc/articles/PMC3721972/ and https://www.ncbi.nlm.nih.gov/pmc/articles/PMC3579943/.
We have added the suggested references, which were very appropriate, and five additional ones.
2)Based on the figure lettering, figure 5 appears distorted. This probably was just an issue with the uploading that will be corrected in the publication quality figure. More of an issue with this figure is the fact that it is not easy to appreciate the features that the authors are trying to emphasize in the figure. Maybe if the authors were able to include some form of quantification or at least a few replicates for comparison it would be easier to visualize what the authors are concluding from these data.
The formatting error within figure 5 has been corrected, we hope it is now possible to clearly see the morphological changes we describe in the text. We took the opportunity to include images of areas away from the wound edge of the scratch, showing similar features.

3)In figure 7, the “away from wound” staining per cell simply visually looks much higher per cell in the 75% BCM than any other treatment. Are the author’s certain the graph and the images are appropriately corresponding in this figure? If there is no mistake, can the authors find a more representative image to display?
Thank you for the suggestion, we have replaced the image for 75% BCM with one from analyzed images that is more representative of the complete data set.

4)In figure 8, I am having trouble observing the phenotype the authors claim exists. Can the authors find a way to quantify this phenotype? Maybe if they did a membrane stain too it would be easier to see in a “merge” that there was more membrane localization in the untreated condition?
Thank you for your comment. The figure now includes higher power images of areas from the original figure merged as well as higher power images of the beta-catenin and nuclei stained. We feel that this combination of representative images makes it easier for reader to see the effects.

5)While I can more easily visually appreciate the claimed phenotype for Figure 9 and even more so for Figure 10 relative to the figure 8 phenotype, a method of quantification for both of these figures would also be appreciated.
Thank you for your comment. We have included a graphical quantification by measuring average particle size showed significant effects of BCM treatment. We have also included higher power images to clearly allow readers to observe the described effects.

6)There is a claimed phenotypic difference in Figure 11 but the graph does not seem to depict any statistically significant differences. No difference in overall expression, quantity but the location and appearance is different?
Thank you for your comment. We have included a graphical quantification of ZO-1 expression by measuring average particle size showed significant effects of BCM treatment. We have also included higher power images to clearly allow readers to observe the described effects.

Minor comments:
1)Change scale bar to white in figure 2A for more accessible viewing.
We have changed the scale bar to white in figure 2A.
2)Change scale bar lettering to a white font in figure 2C for easier reading.
We have changed scale bar lettering to a white font in figure 2C.
3)Scale bar missing for figure 3A
Scale bar included in figure 3A
4)Scale very difficult to read on figure 4A
We have increased the font size and the dimensions of the scale bar on Figure 4A
5)On page 3 lines 42-45, the authors’ statement about it studying biofilm secretions rather than bacterial coat proteins might need better clarification.
We have reworded this sentence for better clarification.
6)There are some grammatical errors such as commas that are not needed and missing commas in many places. I would recommend the authors perform an additional proofreading to this paper and correct these grammatical errors.
Thank you for the suggestion. We have conducted an additional proof reading to improve the grammar and prose and we have had it checked by a professional editor.


Reviewer: 2

Comments to the Author
Interesting in vitro results of the effects of S. aureus biofilm products. The limitation of the study should, however, be discussed such as the use of only one target eukaryotic cell (3T3 mouse fibroblasts) and one bacterial strain. Do human cells behave the same? The authors speculate on the possible selective negative effects on wound edge fibroblasts; but why do the authors think that these cells are more sensitive to the deleterious effect of S. aureus biofilm secretions than cells (e.g., fibroblasts) in the wound bed. Finally, the authors should discuss possible factors that are produced by the biofilm causing cell damages.

Specific comments
1. Abstract page 2, line 15: Please specify etiology of the chronic wound S. aureus was derived from.
We have included additional details on the S. aureus.

2. Page 4, line 52, 60: change ul to µl.
We have corrected this error, thank you.

3. Page 5, line 12: Please indicate seeding density in the scratch experiment.
We have included details in relevant methods section.

4. Page 7, line 18: Please indicate the definition of statistical significance.
We have included details in relevant methods section.

5. Page 7, line 46, 56: Present the magnitude of the effects rather than the p values.
We have been more descriptive in text to be clearer in reporting our findings.

6. Page 11, line 33: Please cite the original finding of senescent fibroblasts in venous leg ulcers (PMID: 10201530).
We thank you for pointing out this important paper. Reference now included.
7. The Discussion is very technical, repetitive of results and too long; please shorten and discuss more general, possible factors that are produced by the biofilm causing cell damages and the limitations of the study.
We have attempted to shorten the discussion and make it more general, highlighting limitations whilst trying not to lengthen it.
